# A Bayesian method and its variational approximation for prediction of genomic breeding values in multiple traits

**DOI:** 10.1186/1471-2105-14-34

**Published:** 2013-01-31

**Authors:** Takeshi Hayashi, Hiroyoshi Iwata

**Affiliations:** 1 Agroinformatics Division, National Agriculture and Food Research Organization, Agricultural Research Center, Kannondai, Tsukuba, Ibaraki, 305-8666, Japan; 2 Department of Agricultural and Environmental Biology, Graduate School of Agricultural and Life Sciences, The University of Tokyo, 1-1-1 Yayoi, Bunkyo, Tokyo, 113-8657, Japan

**Keywords:** Genomic selection, Multiple traits, Bayesian regression, MCMC iteration, Variational approximation

## Abstract

**Background:**

Genomic selection is an effective tool for animal and plant breeding, allowing effective individual selection without phenotypic records through the prediction of genomic breeding value (GBV). To date, genomic selection has focused on a single trait. However, actual breeding often targets multiple correlated traits, and, therefore, joint analysis taking into consideration the correlation between traits, which might result in more accurate GBV prediction than analyzing each trait separately, is suitable for multi-trait genomic selection. This would require an extension of the prediction model for single-trait GBV to multi-trait case. As the computational burden of multi-trait analysis is even higher than that of single-trait analysis, an effective computational method for constructing a multi-trait prediction model is also needed.

**Results:**

We described a Bayesian regression model incorporating variable selection for jointly predicting GBVs of multiple traits and devised both an MCMC iteration and variational approximation for Bayesian estimation of parameters in this multi-trait model. The proposed Bayesian procedures with MCMC iteration and variational approximation were referred to as MCBayes and varBayes, respectively. Using simulated datasets of SNP genotypes and phenotypes for three traits with high and low heritabilities, we compared the accuracy in predicting GBVs between multi-trait and single-trait analyses as well as between MCBayes and varBayes. The results showed that, compared to single-trait analysis, multi-trait analysis enabled much more accurate GBV prediction for low-heritability traits correlated with high-heritability traits, by utilizing the correlation structure between traits, while the prediction accuracy for uncorrelated low-heritability traits was comparable or less with multi-trait analysis in comparison with single-trait analysis depending on the setting for prior probability that a SNP has zero effect. Although the prediction accuracy with varBayes was generally lower than with MCBayes, the loss in accuracy was slight. The computational time was greatly reduced with varBayes.

**Conclusions:**

In genomic selection for multiple correlated traits, multi-trait analysis was more beneficial than single-trait analysis and varBayes was much advantageous over MCBayes in computational time, which would outweigh the loss of prediction accuracy caused by the approximation procedure, and is thus considered a practical method of choice.

## Background

A huge number of genome-wide polymorphisms have recently been elucidated in livestock and crops with the development of sequencing technologies. High-throughput genotyping systems, such as high-density SNP chips containing several tens or hundreds of thousands of genome-wide SNP markers and GBS (genotyping by sequence) [1], have become available to efficiently identify genotypes of individuals for a large number of SNPs at low cost. Consequently, genomic selection [2] is attracting attention as a new breeding technology utilizing the information of genome-wide dense SNP markers. In genomic selection, a model to predict genomic breeding value (GBV) based on genome-wide SNP genotype is firstly constructed using a training population consisting of individuals with both SNP genotypes and phenotypes of a trait and, subsequently, using this model, the GBVs of a trait are predicted for individuals in the tested population, which are selection candidates, based on their SNP genotypes, allowing effective individual selection to be performed without phenotypic records using the predicted GBV. Therefore, genomic selection requires the construction of prediction models being able to accurately relate genotypes of genome-wide SNPs to GBV.

In the original study of genomic selection by Meuwissen et al. [2], two Bayesian methods were presented for construction of prediction models, which were referred to as BaeysA and BayesB and have been used for the studies of genomic selection in their original or modified forms [3,4]. The model of BayesA is equivalent to the Bayesian shrinkage regression (BSR) model [5], where all SNPs are included in the prediction model as covariates and the estimates of SNP effects are shrunk by assuming a normal distribution with mean 0 and SNP-specific variance as prior distributions of SNP effects, resulting in negligible estimates for the effects of many SNPs irrelevant to phenotype, but significantly large estimates for the effects of SNPs contributing to phenotype. On the other hand, the BayesB method is regarded as a variant of Bayesian stochastic search variable selection (BSSVS) [6], where the prior probability, *π*, that a SNP has zero effect and is removed from the model was considered in the model fitting to obtain the best model explaining the phenotypes with a small number of SNP effects. A normal distribution with mean 0 and SNP-specific variance is assumed for the prior distribution of SNP effects in BayesB, as in BayesA, if SNPs are included in the model with probability 1-*π* and, otherwise, both the mean and variance are fixed at 0 with probability *π*.

In BayesA and BayesB, an additional hierarchical structure is induced for this SNP-specific variance, where an inverted chi-square distribution with degree of freedom *ν* and scale parameter *S* is adopted as the prior distribution of the variance. However, only the information of the relevant single SNP can be used for the posterior inference of this SNP-specific variance regardless of the number of genotypes or phenotypes. Due to the insufficient Bayesian learning for the variance, the degree of shrinkage for the estimates of SNP effects is largely influenced by the prior setting for *ν* and *S* in BayesA and BayesB [7]. In BaeysB, the sparseness of the model and the prediction accuracy are also greatly affected by the prior probability, *π*, that a SNP has zero effect, which is treated as a known fixed value. To overcome these drawbacks of BayesA and BayesB, the modified methods such as BayesC and BayesD were proposed. In BayesC, a single variance common to all SNPs is adopted for the prior distribution of SNP effects while BayesD allows *S* to be inferred from the data, given *ν*. Furthermore, the step for inferring *π*, which is given as a fixed value in BayesB, can be incorporated in the procedure of BayesC and BayesD and the corresponding methods are termed BayesC*π* and BayesD*π*, respectively [4].

These Bayesian methods were mainly developed for genomic selection of a single trait. However, actual breeding of animals and plants often aims to simultaneously improve multiple correlated traits. Therefore, joint prediction of GBVs for multiple traits, taking into consideration the correlation structure between traits, is suitable for multi-trait genomic selection, which requires the extension of existing methods for single-trait GBV prediction to multi-trait case. In QTL mapping methods that use similar models to those of genomic selection, some researchers have developed multi-trait models [8-10]. Xu et al. [8] extended the BSR model to a multi-trait case introducing the correlation structure between traits in estimation of QTL effects and other non-genetic effects. Banerjee et al. [9] employed a Bayesian composite model space approach [11,12] for multi-trait QTL mapping, where a variable indicating inclusion or exclusion of each QTL was incorporated for constructing a model with the smallest possible number of QTLs, a similar approach to BSSVS adopted in BayesB, and each trait was allowed to have a different model by assuming the trait-specific effects for QTLs and non-genetic factors that were assumed to be uncorrelated between traits. Meuwissen and Goddard [10] proposed a multi-trait BSSVS model for QTL mapping. These methods can be applied for prediction of multi-trait GBVs in genomic selection. Calus and Veerkamp [13] applied the method proposed in [10] with some modification for multi-trait GBV prediction to compare the accuracy between single-trait prediction and multi-trait prediction in genomic selection.

The computational procedure with MCMC iteration is generally used for the Bayesian methods to estimate parameters in the models, which become complicated models in genomic selection, including a huge number of SNPs as covariates and SNP effects that are estimated as their regression coefficients. The computational burden of MCMC-based Bayesian methods, which requires a long time until convergence when estimating many parameters is huge even in single-trait GBV prediction and would be further increased in the case of multiple traits, thus hindering the MCMC procedure depending on the number of traits to be jointly analyzed. Therefore, it would be necessary to devise a solution for reducing the computational burden of Bayesian methods for multi-trait GBV prediction. So far, some non-MCMC computational procedures for Bayesian methods have been proposed in QTL mapping and genome-wide association study, including EM-algorithm [14] and variational approximation [15,16]. The EM-algorithm was also applied to single-trait GBV prediction, successfully reducing the computational time [17,18].

In this paper, we propose Bayesian methods for multi-trait GBV prediction, in which BSR models allowing variable selection are developed and MCMC procedures for estimating model parameters including SNP effects are described as well as a computationally cost-effective non-MCMC method using variational approximation as an alternative computational procedure. Hereafter, the Bayesian methods based on MCMC iteration and variational approximation are referred to as MCBayes and varBayes, respectively. The multi-trait Bayesian models described here include a Bayesian shrinkage regression (BSR) models that are equivalent to those adopted by BayesA and BayesD when variable selection is not conducted, and the models of BSSVS that are regarded as slightly modified versions of BayesB and BayesD*π* methods when a step of variable selection step is incorporated. We develop computationally-effective variational approximation procedures to construct the posterior distributions of parameters of these Bayesian models.

Using simulated datasets consisting of genotypes of genome-wide dense SNPs and phenotypes of three correlated traits with high and low heritabilities, we investigated the differences in prediction accuracy for each trait between multi-trait analysis, where GBVs of three traits were simultaneously predicted taking the correlation structure between traits into consideration, and single-trait analysis, where each trait was separately predicted for GBV. We also evaluated the prediction accuracy of the varBayes methods in comparison with MCBayes. Moreover, we investigated the performance of multi-trait analysis in simulated data including missing phenotypes.

## Methods

In this section, we describe Bayesian models for multi-trait GBV prediction and computational procedures for Bayesian estimation of the model parameters, including construction of posterior distributions of the parameters using MCMC iteration and variational approximation. Here, we consider the statistical models for BSR and BSSVS, which are shown to be equivalent to BayesD and similar to BayesD*π*, respectively. In these Bayesian models concerned, BSR is regarded as a special version of BSSVS by setting *π* =0, where *π* is a prior probability that a SNP does not contribute to traits; thus, we present the multi-trait GBV prediction models in a unified fashion in terms of BSSVS.

We assume that the number of SNPs genotyped is *N* and a training dataset including *n* individuals with phenotypic records of multiple traits, where the number of traits is denoted by *T*, and SNP genotypes is available for estimating parameters in the prediction model. We also assume that a test dataset consists of individuals with only SNP genotypes, for each of which GBV is predicted. We denote two alleles at each SNP by 0 and 1 and three genotypes by ‘0_0’, ‘0_1’, and ‘1_1’.

### Models for Bayesian stochastic search variable selection in multi-trait genomic selection

We propose the following Bayesian multi-locus linear model for the phenotypes of *T* traits of the *i*th individual, which are denoted as a *T*x1 vector **y**_*i*_ (*i* = 1,2,…,*n*), as a BSSVS model for multi-trait GBV prediction:

(1) $$y_{i}=X_{i}b+\sum_{l=1}^{N}\gamma_{l}u_{\mathrm{il}}g_{l}+e_{i}\text{,}$$

where **b** is a *f ×* 1 vector of non-genetic effects including interceptions of the model with **X**_*i*_ being a *T × f* design matrix linking **b** to the *i*th individual; *u*_*il*_ is a variable indicating the genotype of the *i*th individual at the *l*th SNP taking a value of −1, 0 or 1 corresponding to the genotypes, ‘0_0’, ‘0_1’, or ‘1_1’, respectively; **g**_*l*_ is a *T ×* 1 vector of the effects of the *l*th SNP on the phenotypes of *T* traits; *γ*_*l*_ is a variable indicating the inclusion of the *l*th SNP in the model with 1 or 0 depending on whether or not the *l*th SNP is included in the model; and **e**_*i*_ is a *T ×* 1 vector of residual errors following a *T*-variate normal distribution *N*(**0**, **Σ**_*e*_) with **0** being a *T ×* 1 vector of zeros and **Σ**_*e*_ being a *T*x*T* covariance matrix of **e**_*i*_.

Within the Bayesian framework, prior distributions are assigned to the parameters of the model (1). We assume that the priors of the elements of **b** are the improper uniform distribution over the possible values. The prior probabilities that *γ*_*l*_ =1 and *γ*_*l*_ =0 are presented as 1- *π* and *π*, respectively, considering the prior probability that a SNP does not contribute to the trait and is excluded from the model is *π*. The prior distribution of **g**_*l*_ given *γ*_*l*_ has the form

(2) $$g_{l}|\gamma_{l}\sim \;\{\begin{array}{cc} N\left(0,\Sigma_{\mathrm{gl}}\right) & \left(\gamma_{l}=1\right)\\ \delta \left(0\right) & \left(\gamma_{l}=0\right) \end{array}\;\text{,}$$

where **Σ**_*gl*_ is a *T × T* matrix that is the variance and covariance matrix of **g**_*l*_ given *γ*_*l*_ =1 and *δ* (**0**) is the delta function that concentrates a total mass at zero for all *T* elements of **g**_*l*_. We induce a hierarchical structure for **Σ**_*gl*_ by assigning an inverse Wishart distribution with degree of freedom *ν* and scale parameter **S**, denoted by IW_*T*_(*ν*,**S**), to the prior distribution of **Σ**_*gl*_ :

(3) $$\Sigma_{\mathrm{gl}}\sim {\mathrm{IW}}_{T}\left(v,S\right)\propto |S{|}^{\nu /2}|\Sigma_{\mathrm{gl}}{|}^{-\left(\nu +T+1\right)/2}\exp\left\{-\frac{1}{2}\mathrm{tr}\left(S\Sigma_{\mathrm{gl}}{}^{-1}\right)\right\}$$

Although the SNP effect **g**_*l*_ is zero and irrelevant to **Σ**_*gl*_ when *γ*_*l*_ =0 as shown in (2), we assume that **Σ**_*gl*_ is a priori distributed as given in (3) regardless of *γ*_*l*_. We treat *ν* as a given fixed value but infer **S** from the data within the Bayesian framework. For simplicity, we assume here that **S** is a diagonal matrix with the *k*th diagonal element being *s*_*k*_ , that is **S** = diag(*s*_*k*_) (*k* = 1,2,…,*T*), and we adopt the improper uniform distribution over positive values for a prior distribution of *s*_*k*_. The residual variance and covariance matrix **Σ**_*e*_ is assumed to have a uniform distribution over the positive definite matrices as a prior distribution.

Denoting these parameters of the Bayesian model collectively by **θ**, the prior distribution of **θ** by *p*(**θ**) and observed phenotypes **y**_*i*_ and SNP genotypes *u*_*il*_ (*i* = 1,2,…,*n*;*l* = 1,2,…,*N*) by **Y** and **U**, respectively, we can write the posterior distribution of **θ**, *g*(**θ|***ν*,**Y**, **U**), as

$$g\left(\theta |\nu ,Y,U\right)\propto |\Sigma_{e}{|}^{-n/2}\exp\left(-\frac{1}{2}\sum_{i=1}^{n}y_{i}*'\Sigma_{e}{}^{-1}y_{i}*\right)$$

×∏l=1N{1−π|Σgl|−1/2exp(−12gl'Σgl−1gl)}γlπδ01−γl

(4) $$\times \prod_{l=1}^{N}|S{|}^{\nu /2}|\Sigma_{\mathrm{gl}}{|}^{-\left(\nu +T+1\right)/2}\exp\left(-\frac{1}{2}\mathrm{tr}S\Sigma_{\mathrm{gt}}{}^{-1}\right)$$

from (1), (2) and (3), where **y**_i_* is a residual given by $y_{i}*=y_{i}-X_{i}b-\sum_{l=1}^{N}\gamma_{l}u_{\mathrm{il}}g_{l}.$

Given that **S** is fixed, posterior distribution (4) is equivalent to that of BayesA extended to multi-trait case when *π* = 0 leading to *γ*_*l*_ =1 for all SNPs. When *π* > 0, the selection of SNPs to be included in the model is performed as in BayesB. However, it should be noted that posterior distribution (4) is not equivalent to that of the multi-trait version of BayesB, which supposes that **Σ**_*gl*_ is a zero matrix as well as **g**_*l*_ when *γ*_*l*_ =0, while, in (4), the prior distribution of **Σ**_*gl*_ is assumed to be IW_*T*_(*ν*, **S**) regardless of *γ*_*l*_. The form of posterior distribution (4) allows Gibbs sampling in the MCMC estimation for all parameters including **g**_*l*_ and **Σ**_*gl*_. The full conditional posterior distributions of parameters used in MCBayes are described in Additional file 1.

### Variational approximation procedure for multi-trait Bayesian model

We adopted variational approximation as an alternative to MCMC iteration for constructing marginal posterior distributions of parameters based on joint posterior distribution (4). In the variational approximation procedure, the joint posterior distribution is approximated by the product of functions for subsets of parameters with lower dimension. Briefly, we assume that *g*(**θ|***ν*, **Y**, **U**) is approximated by a function of **θ**, *q*(**θ**), which is factorized as *q*(**θ**) = *q*_1_(**θ**_1_) *q*_2_(**θ**_2_)…*q*_*K*_(**θ**_*K*_), where **θ**_1_, **θ**_2_,… ,**θ**_*K*_ are mutually exclusive subsets of **θ** such that ∪ _*k* = 1_^*K*^**θ**_*k*_ = **θ** and *q*_*k*_ (**θ**_*k*_) (*k* = 1,2,…,*K*) may generally depend on *ν***Y** and **U** although this dependence is omitted here for simplicity. This approximating marginal posterior distribution, *q*_*k*_(**θ**_*k*_), is referred to as the variational posterior of **θ**_*k*_[19]. The form of *q*_*k*_(**θ**_*k*_) is determined such that the Kullback–Leibler divergence between g(**θ|***ν***Y**, **U**) and *q*(**θ|***q*_1_(**θ**_1_) *q*_2_(**θ**_2_)…*q*_*K*_(**θ**_*K*_), *D*(*q*||*g*), is minimized [20], where *D*(*q*||*g*) is defined as

$$D\left(q||g\right)=\int q\left(\theta \right)\log\frac{q\left(\theta \right)}{g\left(\theta |\nu ,Y,U\right)}d\theta$$

It can be shown that *q*_*k*_(**θ**_*k*_) is expressed as

(5) $$q_{k}\left(\theta_{k}\right)=C\exp\left\{E_{-}{{}_{\theta }}_{k}\left[\log\;g\left(\theta |\nu ,Y,U\right)\right]\right\}$$

where *C* is a normalized constant and E_*-*__**θ**__*k*_[.] indicates the expectations of parameters other than **θ**_*k*_ that are calculated with respect to every other parameter’s variational posteriors except *q*_*k*_(**θ**_*k*_) [21].

In the varBayes method that applies the variational approximation procedure to the multi-trait Bayesian model considered here, g(**θ**|*ν*,**Y**, **U**) is assumed to be approximated by a factorized function *q*(**θ**) that is written as

$$q\left(\theta \right)=q\left(b\right)q\left(\Sigma_{e}\right)\prod_{l=1}^{N}\left\{q\left(\gamma_{l},g_{l}\right)q\left(\Sigma {}_{\mathrm{gl}}\right)\right\}q\left(S\right)$$

where we denote all of the variational posteriors for the different parameters in the right-hand side by *q*(.) for simplicity with the understanding that *q*(**b**), *q* (**Σ**_*e*_) and so on take different forms depending on the parameters. The forms of these variational posteriors are derived from (5) in a manner similar to that used for derivation of the full conditional posteriors for the parameters in Gibbs sampling and the computational details are given in Additional file 2. In the following, we will give the variational posteriors for parameters, *γ*_*l*_, **g**_*l*_, **Σ**_*gl*_ (*l* = 1,2,…,*N*), **b**, **Σ**_*e*_ and **S**.

The variational posterior for **b**, *q*(**b**),is a multivariate normal density with mean

(6) Eb=∑i=1nXi'EΣe−1Xi−1∑i=1nXi'EΣe−1yi−∑l=1NuilEγlgl

and variance-covariance matrix

Vb=∑i=1nXi'EΣe−1Xi−1

where *E*(.) indicates the expectation calculated with respect to the variational posteriors of relevant parameters, while *q* (**Σ**_*e*_) is IW_*T*_ (*n-T-*1, **S**_*e*_) with

(7) Se=∑i=1nyi−XiEb−∑l=1NuilEγlglyi−XiEb−∑l=1NuilEγlgl'

from which we obtain

(8) $$\;E\left(\Sigma_{e}{}^{-1}\right)=\left(n-T-1\right)S_{e}{}^{-1}$$

The variational posterior of **Σ**_*gl*_ is represented by IW_*T*_ (*ν*_*gl*_, **S**_*gl*_), where

$$\nu_{\mathrm{gl}}=\nu +E\left(\gamma_{l}\right)$$

and

$$S_{\mathrm{gl}}=E\left(S\right)+E\left(\gamma_{l}g_{l}g_{l}’\right)$$

The expectation of **Σ**_*gl*_^-1^ with respect to this posterior distribution is

(9) $$\begin{array}{cc} \;E\left(\Sigma_{\mathrm{gl}}{}^{-1}\right) & =\nu_{\mathrm{gl}}S_{\mathrm{gl}}{}^{-1}\\ & =\left\{\nu +E\left(\gamma_{l}\right)\right\}\left\{\;E\left(S\right)+E\left(\gamma_{l}\;g_{l}g_{l}’\right)\right\} \end{array}$$

For *γ*_*l*_ and **g**_*l*_, we consider joint distribution for variational posterior *q*(*γ*_*l*_, **g**_*l*_) that is expressed as

(10) qγl,gl∝1−π|Vgl|1/2|EΣl−1|1/2exp12g^l'Vgl−1g^l×ϕgl|g^l,Vglγl×πδ01−γl

where $\varphi \left(g_{l}|{\hat{g}}_{l},V_{\mathrm{gl}}\right)$ is a density function of a multivariate normal distribution $N\left({\hat{g}}_{l},V_{\mathrm{gl}}\right)$ with mean

(11) $$\begin{array}{cc} \;{\hat{g}}_{l}= & {\left\{E\left(\Sigma_{l}{}^{-1}\right)+\sum_{i=1}^{n}u_{\mathrm{il}}{}^{2}E\left(\Sigma_{e}{}^{-1}\right)\right\}}^{-1}E\left(\Sigma_{e}{}^{-1}\right)\sum_{i=1}^{n}u_{\mathrm{il}}\\ & \;\left\{y_{i}-X_{i}E\left(b\right)-\sum_{j\neq l}u_{\mathrm{ij}}E\left(\gamma_{j}g_{j}\right)\right\} \end{array}$$

and variance-covariance matrix

(12) $$V_{\mathrm{gl}}={\left\{E\left(\Sigma_{l}{}^{-1}\right)+\sum_{i=1}^{n}u_{\mathrm{il}}{}^{2}E\left(\Sigma_{e}{}^{-1}\right)\right\}}^{-1}$$

The marginal posterior distribution of *γ*_*l*_ is a binomial distribution with probabilities of *γ*_*l*_ =1 and 0 given by

(13) qγl=1=Eγl=1−π|Vgl|1/2|EΣl−1|1/2exp12g^l'Vgl−1g^l1−π|Vgl|1/2|EΣl−1|1/2exp12g^l'Vgl−1g^l+π

and

qγl=0=1−qγl=1=π1−π|Vgl|1/2|EΣl−1|1/2exp12g^l'Vgl−1g^l+π

The conditional distribution of **g**_*l*_ given *γ*_*l*_ is given by

$$q\left(g_{l}|\gamma_{l}\right)=\left\{\begin{array}{cc} N\left({\hat{g}}_{l},V_{\mathrm{gl}}\right) & \left(\gamma_{l}=1\right)\\ \delta \left(0\right) & \left(\gamma_{l}=0\right) \end{array}\right)$$

Therefore, we obtain

(14) $$\begin{array}{cc} \;E\left(\gamma_{l}g_{l}\right) & =q\left(\gamma_{l}=1\right)E\left(g_{l}|\gamma_{l}=1\right)+q\left(\gamma_{l}=0\right)E\left(g_{l}|\gamma_{l}=0\right)\\ & =E\left(\gamma_{l}\right){\hat{g}}_{l} \end{array}$$

and

(15) Eγlglgl’=Eγlg^lg^l'+Vgl

From (4), the variational posterior of **S** is a *T*-variate Wishart distribution with a scale matrix **Σ**_*S*_ and degree of freedom *ν*_*s*_, W_*T*_(*ν*_*s*_, **Σ**_*S*_), where $\Sigma {}_{S}=\left\{\sum_{l=1}^{N}E\left(\Sigma_{\mathrm{gl}}^{-1}\right)\right\}-1$ and *ν*_*s*_ = *Nν* + *T* + 1, and the expectation of **S** is expressed as

(16) $$\begin{array}{cc} \;E\left(S\right) & =\nu_{s}\Sigma {}_{S}\\ & =\left(\mathrm{N\nu}+T+1\right){\left\{\sum_{l=1}^{N}E\left(\Sigma_{\mathrm{gl}}{}^{-1}\right)\right\}}^{-1} \end{array}$$

As outlined above, a well-known probability distribution, such as normal, inverse Wishart and so on, is assigned to the variational posterior of each parameter, which is characterized by the expectations of the functions of other parameters, taken with respect to their variational posteriors. The relationships between these expectations are given by (6), (8), (9), (13), (14), (15) and (16), from which the expectations can be calculated with numerical iterations to obtain the variational posteriors.

Moreover, the prior probability for a SNP to have zero effect, *π*, can be treated as a variable parameter to be inferred from the data as in BayesC*π* and BayesD*π* when 0 < *π* < 1. Here, we assume a uniform prior on 0 < *π* < 1 to obtain a Beta distribution, B(*a*,*b*), as a variational posterior from (4), where $a=N-\sum_{l=1}^{N}E(\gamma_{1})+1$ and $b=\sum_{l=1}^{N}E(\gamma_{1})+1$, with the expectation

(17) $$\begin{array}{cc} \;E\left(\pi \right) & =a/\left(a+b\right)\\ & =\left\{N-\sum_{l=1}^{N}E\left(\gamma_{l}\right)+1\right\}/\left(N+2\right) \end{array}$$

Accordingly, the variational posteriors of the other parameters are modified with *π* substituted by *E*(*π*) when *π* is involved in the Bayesian inference.

### Treatment of missing phenotypes

In the phenotypic records for multiple traits, it is common for trait values to be partially missing in some individuals. Missing phenotypes of a trait in individuals can be inferred with the observed phenotypes of other traits in the same individuals. The step for inferring missing phenotypes can be implemented in the MCBayes and varBayes procedures as described below.

When there are missing phenotypes in an individual, the residual vector of the individual, **e** in model (1), is partitioned into components **e**_o_ and **e**_m_ corresponding to observed and missing phenotypes, respectively. Following [22], **e**_m_ can be sampled from the following normal distribution,

(18) $$e_{m}\sim N\left(\Sigma_{\mathrm{mo}}\Sigma_{\mathrm{oo}}{}^{-1}e_{o},\Sigma_{\mathrm{mm}}-\Sigma_{\mathrm{mo}}\Sigma_{\mathrm{oo}}{}^{-1}\Sigma_{\mathrm{mo}}’\right)$$

where **Σ**_mm_, **Σ**_mo_ and **Σ**_oo_ indicate the partition of the residual variance-covariance matrix **Σ**_*e*_ corresponding to **e**_m_ and **e**_o_. Accordingly, **e**_m_ is drawn with Gibbs sampling in MCBayes while it is obtained as E(**Σ**_mo_)E(**Σ**_oo_)^-1^**e**_o_* in varBayes, where, denoting the component corresponding to the missing traits by subscript ‘o’, **e**_o_* is written as

$$e_{o}*=y_{o}-X_{o}E\left(b\right)-\sum_{l=1}^{N}u_{\mathrm{il}}E\left(\gamma_{l}g_{ol}\right)$$

and the expectations can be calculated with the variational posteriors of **Σ**_*e*_, **b** and **g**_*l*_ . Missing phenotypes are inferred as the sum of the estimates of **b**, **g**_*l*_ and **e**_m_, which are used for the construction of the prediction model.

### Simulation experiments

We simulated datasets to evaluate the accuracy of the predicted GBVs using the proposed Bayesian methods, MCBayes and varBayes, for multiple traits. In generating the datasets, three traits, denoted as A, B and C, were considered. The simulation of population and genome was carried out following [3] where a single trait was treated, while multiple traits were generated through a modified approach. The simulated population, genotypes and phenotypes are described in the following.

Populations with an effective population size of 100 were maintained by random mating for 1000 generations to attain mutation drift balance and LD between SNPs and QTLs. In generation 1001 and 1002, the population size was increased to 1000. The population in the 1001st generation was treated as a training population, where the phenotypes of three traits and SNP genotypes of the individuals were simulated and analyzed to estimate the SNP effects in the model. The phenotype of each trait for each individual in the 1001st generation was given as the sum of QTL effects over the polymorphic QTLs and environmental effects, which were sampled as described later. For simplicity, no other fixed effects were assumed. The population in the 1002nd generation was used as a test population, where the individuals were only genotyped for SNP markers without phenotypic records and GBVs of three traits were predicted for each individual using a model with SNP effects estimated based on the training population in the 1001st generation. The true breeding value (TBV) of the individual in the 1002nd generation was also simulated as the sum of QTL effects corresponding to the QTL genotype for each trait and used for evaluating the accuracy of predicted GBV, but was regarded as unknown and unavailable in the estimation of SNP effects in the models. Accuracy was measured based on the correlation between the TBV and predicted GBV, *r*_TBV,pGBV_, for each trait and regression of TBV on predicted GBV, *b*_TBV,pGBV_, was also obtained for assessing the bias of the prediction.

The genome was assumed to consist of 10 chromosomes each 100cM in length. Two scenarios were considered for the number of available SNP markers and the datasets under these two scenarios were denoted as Data I and Data II. In Data I, 101 marker loci were located every 1cM on each chromosome for a total of 1010 markers on a genome. In Data II, 1010 equidistant marker loci were located on each chromosome for a total of 10100 markers. We assumed that 100 equidistant QTLs were located on each chromosome such that a QTL was in the middle between two marker loci in both Data I and Data II. Therefore, there were a total of 1000 QTLs located on a whole genome. The mutation rates assumed per locus per meiosis were 2.5 × 10^-3^ and 5.0 × 10^-5^ for the marker locus and QTL, respectively. At least one mutation occurred in the most of the marker loci with a high mutation rate during the simulated generations. In the marker loci experiencing more than one mutation, the mutation remaining at the highest minor allele frequency (MAF) was regarded as visible, whereas the others were ignored, which resulted in the marker loci having two alleles similar to SNP markers. Although the mutation rate for QTL was assumed 2.5 × 10^-5^ in the simulation for a single trait conducted in [3], we here doubled it for generating TBVs of multiple traits for the reason as described below.

The polymorphic QTLs at which mutation occurred were used to simulate the three traits, A, B and C, the heritabilities of which, denoted by *h*_A_^2^, *h*_B_^2^ and *h*_C_^2^, respectively, were assumed to be *h*_A_^2^ = 0.8, *h*_B_^2^ = 0.1 and *h*_C_^2^ = 0.1. We divided all polymorphic QTLs into four groups, Group1, Group2, Group3 and Group4, according to the causal relationship with traits, where Group1 was a group of QTLs affecting both traits A and B, Group2 was a group of QTLs affecting only trait A, Group3 was a group of QTLs affecting only trait B and Group4 was a group of QTLs affecting only trait C. Therefore, it was assumed that QTLs of Group1 had pleiotropic effects on traits A and B while QTLs of other groups affected only a single trait. Each polymorphic QTL was randomly assigned to one of these groups, that is, to Group1, Group2, Group3 and Group4 with respective probabilities 0.4, 0.1, 0.1 and 0.4. Therefore, 80% of the QTL loci affecting trait A were shared by trait B on average and whereas all the QTL loci affecting trait C influence neither traits A nor B. In this setting of multi-trait case, the mutation rate of QTL was increased to 5.0 × 10^-5^ from 2.5 × 10^-5^, the mutation rate adopted in [3] for generating a single trait, to retain the number of QTL per trait.

The pleiotropic effects of each QTL in Group1 were assumed to be correlated between traits A and B with correlation coefficient of 0.9. Consequently, genetic correlations between traits A and B, between A and C and between B and C, denoted as *ρ*_GAB_, *ρ*_GAC_ and *ρ*_GBC_, respectively, were *ρ*_GAB_ = 0.72 and *ρ*_GAC_ = *ρ*_GBC_ = 0 on average although the values of correlation coefficients were somewhat fluctuated in each data generation. This setting of traits was adopted to investigate how the prediction accuracy was increased for a low-heritability trait (trait B) by simultaneous analysis for multiple traits including a correlated high-heritability trait (trait A).

The effects of QTL alleles were sampled from gamma distributions independently for each QTL. Pleiotropic effects of QTL alleles in Group1 were determined for traits A and B by generating two correlated gamma random variables, *x* and *y*, with the correlation coefficient of 0.9, and assigning them with positive or negative values with equal probabilities. The effects of QTL alleles in the other groups, which affected each single trait only, was determined by sampling a gamma random variable *z* and by similarly assigning it with the positive or negative sign. We generated these three random variables *x*, *y* and *z* such that their marginal distributions were the same gamma distribution with scale parameter 0.4 and shape parameter 1.66, Gamma(0.4,1.66). For obtaining correlated variables *x* and *y*, we generated three independent gamma variables, *x*_1_, *x*_2_ and *x*_3_, which were sampled from Gamma(0.36,1.66), Gamma(0.04,1.66) and Gamma(0.04,1.66), respectively, and determined the values of *x* and *y* as *x* = *x*_1_ + *x*_2_ and *y* = *x*_1_ + *x*_3_. It can be shown that *x* and *y* had a correlation coefficient of 0.9 and the same marginal distribution Gamma(0.4,1.66) [23].

The environmental correlation coefficients between three traits were denoted by *ρ*_EAB_, *ρ*_EAC_ and *ρ*_EBC_, respectively, and assumed as *ρ*_EAB_ = 0.1, *ρ*_EAC_ = 0.2 and *ρ*_EBC_ = 0.3. The environmental effects were sampled from a trivariate normal distribution with a mean vector **0** and a variance-covariance matrix **R**_E_, where

$$R_{E}=\left(\begin{array}{ccc} \sigma_{\mathrm{EA}}{}^{2} & \rho_{\mathrm{EAB}}\sigma_{\mathrm{EA}}\sigma_{\mathrm{EB}} & \rho_{\mathrm{EAC}}\sigma_{\mathrm{EA}}\sigma_{\mathrm{EC}}\\ \rho_{\mathrm{EAB}}\sigma_{\mathrm{EA}}\sigma_{\mathrm{EB}} & \sigma_{\mathrm{EB}}{}^{2} & \rho_{\mathrm{EBC}}\sigma_{\mathrm{EB}}\sigma_{\mathrm{EC}}\\ \rho_{\mathrm{EAC}}\sigma_{\mathrm{EA}}\sigma_{\mathrm{EC}} & \rho_{\mathrm{EBC}}\sigma_{\mathrm{EB}}\sigma_{\mathrm{EC}} & \sigma_{\mathrm{EC}}{}^{2} \end{array}\right)$$

with *σ*_EA_^2^, *σ*_EB_^2^ and *σ*_EC_^2^ indicating environmental variances of three traits. Environmental variance of trait A was given by *σ*_EA_^2^ = (1/*h*_A_^2^-1)*σ*_GA_^2^ with its heritability *h*_A_^2^ and genetic variance *σ*_GA_^2^, which was variance of TBV of trait A, and those of traits B and C were obtained similarly. The environmental effects were added to TBVs which were given by the sum of QTL effects to determine phenotypic values of three traits for individuals in the 1001st generation.

In Data I, 100 replicated datasets were simulated while Data II consisted of 20 replicated datasets due to the larger number of SNPs. Each of replicated datasets included records of phenotypes of three traits and genotypes of SNPs for the training population (1001st generation) and only SNP genotypes for the test population (1002nd generation). To simulate the situation of missing phenotypes, we generated additional datasets by deleting the phenotypic records of some traits for some individuals in the 100 replicated training datasets of Data I. These 100 replicated datasets were referred to as Data III, where the phenotypic records of traits A, B and C were respectively deleted for individuals of *i* = 801–1000, individuals of *i* = 1-500 and individuals of *i* = 201-700 in 1000 individuals (*i* = 1-1000) of the 1001st generation of Data I. Therefore, in Data III, the prediction model for GBVs was constructed with a training dataset consisting of the phenotypes of 800, 500 and 500 individuals for traits A, B and C, respectively, where only 100 individuals (*i* = 701-800) had phenotypic records of all three traits, and 1000 individuals in the 1002nd generation were predicted for GBV based on the genotypes of 1010 markers. The setting of non-zero environmental correlations, i.e., *ρ*_EAB_ = 0.1, *ρ*_EAC_ = 0.2 and *ρ*_EBC_ = 0.3, was adopted here to assess the benefit from the estimation process of missing phenotypes implemented in multi-trait analyses for the prediction accuracy, where the information of environmental covariance between observed and missing phenotypes was utilized as in (18).

Each replicated dataset in Data I, Data II and Data III was analyzed using the proposed methods for multiple traits, MCBayes and varBayes, to construct the GBV prediction model in the1001st generation and investigate *r*_TBV,pGBV_ and *b*_TBV, pGBV_ for each trait in the 1002nd generation. For a comparison with conventional single-trait GBV prediction, the same datasets were also analyzed with the single-trait setting of MCBayes where three traits were separately treated without considering the correlation structure between traits.

We conducted cross-validation as well to evaluate the prediction performance within a population in the 1001st generation without the 1002nd generation since the techniques of cross-validation have commonly been used for evaluation of the accuracy in the studies of genomic selection with the actual datasets of animals [24-26] and plants [27-29], where the prediction accuracy of the model for the unobserved future samples was of concern. We applied 10-fold cross-validation. In brief, we randomly split a population of 1000 individuals in the 1001st generation into ten subpopulations with each size 100. By using a single subpopulation used as a test set and the remaining nine subpopulations as a training set to construct prediction model, the prediction accuracy and bias were assessed for the test set. This process was repeated ten times until all single subpopulations were used as test sets exactly once. We averaged the prediction accuracy and bias over ten repetitions to evaluate the prediction performance in each dataset. Because of much computational burden with MCMC analysis for repeated model constructions in cross-validation, evaluation with 10-fold cross-validation was carried out for Data I only, where mean of the prediction accuracies and biases in 100 datasets were obtained as *r*_TBV,pGBV_ and *b*_TBV, pGBV_ for each trait and for each prediction method. In the process of cross-validation, we also investigated the correlation between predicted GBV and the phenotypic value, *r*_y,pGBV_, in place of TBV, and regression of predicted GBV on phenotypic value, *b*_y, pGBV_, for each trait.

Two settings for the prior probability that a SNP has zero effect, *π*, were adopted in model construction, i.e., *π* was fixed at 0 or *π* was variable over the range 0 < *π* <1 and inferred from the data. The values of hyperparameter *ν*, degree of freedom of prior inverse Wishart distribution of **Σ**_*gl*_, were set to 5.0 and 3.2 corresponding to *π* =0 and 0 < *π* <1 after preliminary analyses to evaluate the effects of the values of *ν* on prediction accuracy over 3 < *ν* <6 although the accuracy was little affected by the values of *ν*.

In the MCMC iteration of MCBayes, we repeated 11000 cycles including a burn-in period of the first 1000 cycles. The values of parameters were sampled every 10 cycles to obtain the posterior means that were used to determine a prediction model for each generated dataset. In the method of varBayes, we adopted the criterion $|{\hat{\theta }}^{*}-\hat{\theta }{|}^{2}/|{\hat{\theta }}^{*}{|}^{2}$ < 10^-8^ for convergence in numerical iteration for computing the expectations of parameters, where ${\hat{\theta }}^{*}$ and $\hat{\theta }$ are the current and previous value of the expectations of parameters. When this criterion was satisfied, the computational procedure with variational approximation was regarded as converged.

## Results

We evaluated the accuracy and bias of the predicted GBVs, *r*_TBV,pGBV_ and *b*_TBV, pGBV_, obtained by the proposed methods for genomic selection of multiple traits including MCBayes and varBayes in 100 simulated datasets of Data I and Data III and in 20 datasets of Data II in comparison with the prediction accuracy with single-trait MCBayes analysis. The means of accuracies and biases of prediction evaluated using a test population in the 1002nd generation are summarized in Table 1, Table 2 and Table 3 for Data I, Data II and Data III, respectively. In all of datasets, MCBayes provided higher prediction accuracy than varBayes in multi-trait analysis (Tables 1, 2 and 3). In Data I, *r*_TBV,pGBV_ obtained with multi-trait MCBayes analysis was 0.788 (0.051), 0.581 (0.103) and 0.453 (0.090) for traits A, B and C, respectively, when *π* was fixed at 0, where standard errors were given in parenthesis here and hereafter, while that was 0.753 (0.060), 0.580 (0.117) and 0.364 (0.137) when *π* was varied and inferred. In Data II with the number of SNPs increased to 10100, *r*_TBV,pGBV_ with multi-trait MCBayes analysis was higher at 0.902 (0.032), 0.706 (0.103) and 0.519 (0.097) for traits A, B and C, respectively, when *π* was fixed at 0 while that was 0.868 (0.047), 0.731 (0.120) and 0.401 (0.182) when *π* was inferred. With multi-trait varBayes analysis, *r*_TBV,pGBV_ was 0.754 (0.061), 0.570 (0.113) and 0.383 (0.117) for traits A, B and C with *π* =0 and that was 0.716 (0.070), 0.548 (0.122) and 0.347 (0.131) with *π* variable in Data I while that was 0.859 (0.049) , 0.656 (0.110) and 0.438 (0.074) with *π* =0 and 0.838 (0.061) , 0.678 (0.140) and 0.330 (0.157) with *π* variable in Data II. The prediction with multi-trait MCBayes was almost unbiased in Data I, where *b*_TBV,pGBV_ was near 1, but was biased in Data II for traits B and C (Tables 1 and 2). The analysis with varBayes showed greater bias than with MCBayes.

**Table 1:** Accuracy and bias of predicted GBVs in Data I

**Method**			**Trait A**	**Trait B**	**Trait C**
MCBayes	*π* = 0	*r*_TBV,pGBV_	0.788 ± 0.051	0.581 ± 0.103	0.453 ± 0.090
		*b*_TBV,pGBV_	0.994 ± 0.038	1.048 ± 0.264	1.00 ± 0.370
	0 < *π* <1	*r*_TBV,pGBV_	0.753 ± 0.060	0.580 ± 0.117	0.364 ± 0.137
		*b*_TBV,pGBV_	1.070 ± 0.064	1.149 ± 0.340	1.016 ± 0.364
varBayes	*π* = 0	*r*_TBV,pGBV_	0.754 ± 0.061	0.570 ± 0.113	0.383 ± 0.117
		*b*_TBV,pGBV_	1.054 ± 0.051	0.994 ± 0.233	0.899 ± 0.247
	0 < *π* <1	*r*_TBV,pGBV_	0.716 ± 0.070	0.548 ± 0.122	0.347 ± 0.131
		*b*_TBV,pGBV_	0.894 ± 0.054	0.834 ± 0.186	0.636 ± 0.202
single-trait	*π* =0	*r*_TBV,pGBV_	0.783 ± 0.051	0.469 ± 0.083	0.455 ± 0.076
(MCBayes)		*b*_TBV,pGBV_	0.978 ± 0.037	1.020 ± 0.301	0.970 ± 0.259
	0 < *π* <1	*r*_TBV,pGBV_	0.778 ± 0.050	0.491 ± 0.114	0.483 ± 0.101
		*b*_TBV,pGBV_	1.089 ± 0.054	1.110 ± 0.634	1.061 ± 0.338

Averages and standard errors based on 100 replicates of simulated data are listed for prediction accuracy, *r*_pGBV,TBV_, and bias, *b*_pGBV,TBV_, of each trait. For the prior probability that a SNP has zero effect, ***π***, we considered two settings, in which ***π*** was fixed at 0, meaning the inclusion of all SNPs in the model, and ***π*** was varied over 0 < ***π*** <1 and inferred from the data.

**Table 2:** Accuracy and bias of predicted GBVs in Data II

**Method**			**Trait A**	**Trait B**	**Trait C**
MCBayes	*π* = 0	*r*_TBV,pGBV_	0.902 ± 0.032	0.706 ± 0.103	0.519 ± 0.097
		*b*_TBV,pGBV_	0.998 ± 0.034	0.902 ± 0.111	0.796 ± 0.179
	0 < *π* <1	*r*_TBV,pGBV_	0.868 ± 0.047	0.731 ± 0.120	0.401 ± 0.182
		*b*_TBV,pGBV_	1.092 ± 0.093	1.189 ± 0.199	1.198 ± 0.553
varBayes	*π* = 0	*r*_TBV,pGBV_	0.859 ± 0.049	0.656 ± 0.110	0.438 ± 0.074
		*b*_TBV,pGBV_	1.059 ± 0.065	0.799 ± 0.105	0.724 ± 0.111
	0 < *π* <1	*r*_TBV,pGBV_	0.838 ± 0.061	0.678 ± 0.140	0.330 ± 0.157
		*b*_TBV,pGBV_	0.983 ± 0.034	0.851 ± 0.138	0.562 ± 0.155
single-trait	*π* =0	*r*_TBV,pGBV_	0.884 ± 0.039	0.485 ± 0.086	0.493 ± 0.089
(MCBayes)		*b*_TBV,pGBV_	0.974 ± 0.035	0.766 ± 0.113	0.766 ± 0.113
	0 < *π* <1	*r*_TBV,pGBV_	0.843 ± 0.044	0.597 ± 0.120	0.601 ± 0.109
		*b*_TBV,pGBV_	1.562 ± 0.261	1.787 ± 0.431	1.832 ± 0.565

Averages and standard errors based on 20 replicates of simulated data are listed for prediction accuracy, *r*_pGBV,TBV_, and bias, *b*_pGBV,TBV_, of each trait. For the settings of the prior probability that a SNP has zero effect, *π*, see Table 1.

**Table 3:** Accuracy and bias of predicted GBVs in Data III

**Method**			**Trait A**	**Trait B**	**Trait C**
MCBayes	*π* = 0	*r*_TBV,pGBV_	0.766 ± 0.058	0.500 ± 0.127	0.322 ± 0.082
		*b*_TBV,pGBV_	0.977 ± 0.048	0.998 ± 0.356	0.967 ± 0.773
	0 < *π* <1	*r*_TBV,pGBV_	0.723 ± 0.069	0.503 ± 0.141	0.202 ± 0.119
		*b*_TBV,pGBV_	1.065 ± 0.076	1.195 ± 0.530	0.799 ± 0.523
varBayes	*π* = 0	*r*_TBV,pGBV_	0.726 ± 0.072	0.447 ± 0.131	0.261 ± 0.134
		*b*_TBV,pGBV_	0.984 ± 0.052	0.582 ± 0.241	0.383 ± 0.181
	0 < *π* <1	*r*_TBV,pGBV_	0.679 ± 0.081	0.387 ± 0.115	0.228 ± 0.112
		*b*_TBV,pGBV_	0.840 ± 0.068	0.389 ± 0.132	0.240 ± 0.110
single-trait	*π* =0	*r*_TBV,pGBV_	0.760 ± 0.058	0.345 ± 0.070	0.336 ± 0.068
(MCBayes)		*b*_TBV,pGBV_	0.965 ± 0.047	0.931 ± 0.368	0.969 ± 0.510
	0 < *π* <1	*r*_TBV,pGBV_	0.758 ± 0.057	0.362 ± 0.105	0.354 ± 0.101
		*b*_TBV,pGBV_	1.086 ± 0.068	1.455 ± 1.401	1.251 ± 1.310

Averages and standard errors based on 100 replicates of simulated data are listed for prediction accuracy, *r*_pGBV,TBV_, and bias, *b*_pGBV,TBV_, of each trait. For the settings of prior probability that a SNP has zero effect, *π*, see Table 1.

In single-trait analysis where MCBayes was applied for a single-trait model, *r*_TBV,pGBV_ was comparable with that of multi-trait analysis for high-heritability trait A while it was significantly decreased for low-heritability trait B which was highly correlated with trait A (Table 1 and Table 2). For trait C which had same heritability as trait B but was genetically independent of trait A (*ρ*_GAC_ = 0.0), multi-trait MCBayes analysis gave the accuracy comparable to single-trait MCBayes analysis when *π* =0 while, when *π* was varied and inferred, single-trait analysis showed considerably higher accuracy than multi-trait MCBayes and varBayes analyses as shown in Table 1 and Table 2. The prediction with single-trait analysis was almost unbiased In Data I, but that was likely to be biased in Data II with *b*_TBV,pGBV_ much deviated from 1 especially when *π* was varied and inferred.

In Data III with missing phenotypes, which was derived from Data I by removing some phenotypic records, *r*_TBV,pGBV_ was decreased for all traits in all methods due to the smaller sample size in comparison with Data I (Table 3). The rate of decrease in *r*_TBV,pGBV_ was greater for traits B and C than A as the greater reduction of the sample size was made in B and C. Multi-trait MCBayes analysis provided higher prediction accuracy than multi-trait varBayes analysis for all traits except for trait C with*π* varied and inferred. For trait B, multi-trait analysis showed higher prediction accuracy than single-trait analysis, but gave less accurate prediction for trait C. For the bias of predicted GBV, *b*_TBV, pGBV_, the MCBayes analysis with *π* =0 was almost unbiased for both multi-trait and single-trait analyses, where *b*_TBV, pGBV_ ranged 0.931 to 0.998, although the standard error of *b*_TBV, pGBV_ was increased for low-heritability traits B and C, for which the MCBayes analyses with *π* varied and varBayes showed much biased prediction.

We listed *r*_TBV, pGBV_ and *b*_TBV, pGBV_ obtained by cross-validation conducted in a population of the 1001st generation of Data I as well as *r*_y,pGBV_ and *b*_y,pGBV_, correlation between predicted GBV and phenotype and regression of phenotype on predicted GBV, in Table 4. The prediction accuracy evaluated with cross-validation was considerably higher than that evaluated with a test population in the next generation, with the prediction bias being similarly for both evaluated with cross-validation and use of a test population. The relative merits in performance of prediction between the methods were also similar for both evaluations. It was shown in Table 4 that the relational expression, *r*_TBV,pGBV_ = *r*_y,pGBV_/*h*[30], held with *h* being a square-root of heritability and that *b*_TBV,pGBV_ was almost the same as *b*_y,pGBV_.

**Table 4:** Accuracy and bias of predicted GBVs evaluated with cross-validation in Data I

**Method**			**Trait A**	**Trait B**	**Trait C**
MCBayes	*π* = 0	*r*_TBV,pGBV_	0.832 **±** 0.039	0.611 **±** 0.095	0.501 **±** 0.083
		*b*_TBV,pGBV_	1.016 **±** 0.022	1.072 **±** 0.231	1.052 **±** 0.341
		*r*_y,pGBV_	0.741 **±** 0.037	0.191 **±** 0.045	0.160 **±** 0.050
		*b*_y,pGBV_	1.013 **±** 0.015	1.062 **±** 0.125	1.039 **±** 0.130
	0 < *π* <1	*r*_TBV,pGBV_	0.791 **±** 0.048	0.603 **±** 0.112	0.390 **±** 0.127
		*b*_TBV,pGBV_	1.132 **±** 0.064	1.210 **±** 0.303	1.180 **±** 0.379
		*r*_y,pGBV_	0.705 **±** 0.045	0.191 **±** 0.046	0.121 **±** 0.060
		*b*_y,pGBV_	1.131 **±** 0.065	1.210 **±** 0.170	1.119 **±** 0.373
varBayes	*π* = 0	*r*_TBV,pGBV_	0.813 **±** 0.049	0.620 **±** 0.108	0.470 **±** 0.118
		*b*_TBV,pGBV_	1.080 **±** 0.048	0.994 **±** 0.157	0.963 **±** 0.201
		*r*_y,pGBV_	0.722 **±** 0.047	0.187 **±** 0.056	0.143 **±** 0.058
		*b*_y,pGBV_	1.072 **±** 0.049	0.945 **±** 0.195	0.931 **±** 0.289
	0 < *π* <1	*r*_TBV,pGBV_	0.779 **±** 0.059	0.593 **±** 0.111	0.423 **±** 0.123
		*b*_TBV,pGBV_	0.944 **±** 0.040	0.816 **±** 0.139	0.662 **±** 0.153
		*r*_y,pGBV_	0.690 **±** 0.056	0.180 **±** 0.055	0.125 **±** 0.062
		*b*_y,pGBV_	0.935 **±** 0.039	0.787 **±** 0.166	0.626 **±** 0.255
single-trait	*π* = 0	*r*_TBV,pGBV_	0.826 **±** 0.040	0.515 **±** 0.073	0.505 **±** 0.074
(MCBayes)		*b*_TBV,pGBV_	0.997 **±** 0.023	1.073 **±** 0.270	1.030 **±** 0.303
		*r*_y,pGBV_	0.735 **±** 0.039	0.159 **±** 0.045	0.162 **±** 0.044
		*b*_y,pGBV_	0.993 **±** 0.012	1.071 **±** 0.162	1.055 **±** 0.222
	0 < *π* <1	*r*_TBV,pGBV_	0.821 **±** 0.039	0.531 **±** 0.099	0.522 **±** 0.094
		*b*_TBV,pGBV_	1.131 **±** 0.046	1.265 **±** 0.555	1.192 **±** 0.405
		*r*_y,pGBV_	0.731 **±** 0.037	0.164 **±** 0.051	0.164 **±** 0.048
		*b*_y,pGBV_	1.127 **±** 0.043	1.249 **±** 0.482	1.205 **±** 0.457

Averages and standard errors evaluated with 10- fold cross-validation are listed based on 100 replicates of simulated data in Data I are listed for prediction accuracy, *r*_pGBV,TBV_, and bias, *b*_pGBV,TBV_, as well as correlation between phenotypic value and predicted GBV, *r*_y,pGBV_, and regression of phenotypic value on predicted GBV, *b*_y,pGBV_ , of each trait. For the settings of prior probability that a SNP has zero effect, *π*, see Table 1.

Correlation coefficients between predicted GBVs of trait A, B and C were listed in Table 5 for multi-trait MCBayes and varBayes analyses and single-trait MCBayes analysis in all datasets as well as the correlation coefficients between simulated breeding values (TBVs) of three traits in the 1002nd generation. Although the correlation of simulated breeding values between traits A and B was expected to be 0.72, the actual correlations obtained were biased upwards: 0.755 (0.133) and 0.735 (0.166) in Data I (III) and Data II, respectively. Multi-trait analysis could better estimate these correlations compared to single-trait analysis as seen in the correlation between predicted GBVs between traits A and B. For trait C which was genetically independent of A and B, the correlations between predicted GBVs for traits A and C and traits B and C did not significantly deviate from zero (Table 5).

**Table 5:** Correlations between predicted GBVs for trait A, B and C

**Data**	**Method**	**A-B (0.72)**	**A-C (0.0)**	**B-C (0.0)**
I	MCBayes *π* = 0	0.588 ± 0.181	−0.071 ± 0.175	−0.091 ± 0.199
	0 < *π* <1	0.699 ± 0.188	−0.057 ± 0.250	−0.076 ± 0.301
	varBayes *π* = 0	0.688 ± 0.184	−0.100 ± 0.241	−0.077 ± 0.279
	0 < *π* <1	0.644 ± 0.169	−0.053 ± 0.192	−0.058 ± 0.247
	Single-trait (MCBayes) *π* = 0	0.446 ± 0.132	0.058 ± 0.096	0.096 ± 0.121
	0 < *π* <1	0.452 ± 0.183	0.035 ± 0.090	0.060 ± 0.111
	Simulated BV	0.755 ± 0.133	0.003 ± 0.054	0.004 ± 0.060
II	MCBayes *π* = 0	0.606 ± 0.197	−0.129 ± 0.113	−0.071 ± 0.140
	0 < *π* <1	0.721 ± 0.222	−0.161 ± 0.149	−0.128 ± 0.179
	varBayes *π* = 0	0.614 ± 0.206	−0.176 ± 0.135	−0.090 ± 0.179
	0 < *π* <1	0.671 ± 0.210	−0.153 ± 0.168	−0.047 ± 0.193
	Single-trait (MCBayes) *π* = 0	0.394 ± 0.115	0.020 ± 0.075	0.072 ± 0.111
	0 < *π* <1	0.479 ± 0.206	−0.018 ± 0.084	0.022 ± 0.094
	Simulated BV	0.735 ± 0.166	−0.031 ± 0.054	−0.012 ± 0.041
III	MCBayes *π* = 0	0.530 ± 0.195	−0.032 ± 0.223	−0.037 ± 0.238
	0 < *π* <1	0.662 ± 0.208	−0.014 ± 0.326	−0.013 ± 0.369
	varBayes *π* = 0	0.555 ± 0.194	−0.028 ± 0.218	−0.023 ± 0.248
	0 < *π* <1	0.455 ± 0.167	−0.001 ± 0.158	0.021 ± 0.190
	Single-trait (MCBayes) *π* = 0	0.315 ± 0.106	0.029 ± 0.105	0.080 ± 0.134
	0 < *π* <1	0.323 ± 0.138	0.024 ± 0.097	0.057 ± 0.130

Averages and standard errors are listed based on 100 replicates of simulated data in Data I and Data III and 20 replicates in Data II. Simulated BV indicates simulated breeding values, where expected correlations are 0.72, 0.0 and 0.0 for trait-pairs A-B, A-C and B-C as listed in parentheses. In Data III, correlations between simulated breeding values are the same as those in Data I.

## Discussion

In this study, we proposed Bayesian methods for simultaneously predicting GBVs for multiple traits, where two computational procedures were devised using MCMC iteration and variational approximation, referred to as MCBayes and varBayes, respectively. A Bayesian model for simultaneously analyzing multiple traits was obtained by extending a Bayesian model for single-trait genomic selection proposed by [2] and [4] to multi-trait case. We introduced the prior probability that a SNP has zero effect, *π*, and accordingly, MCBayes with *π* fixed at 0, meaning that all SNPs are included in a model as covariates, constructs a model for multi-trait GBV prediction in a similar manner to BayesD. On the other hand, the MCMC procedure of MCBayes with *π* variable and inferred from the data is not exactly the same as that of BayesD*π*, where a prior distribution of the variance and covariance matrix of SNP effects, **Σ**_*gl*_, is assumed to be independent of whether the SNP is included (*γ*_*l*_ = 1) or excluded(*γ*_*l*_ = 0) from the model in MCBayes, as seen in (4), while it is dependent on*γ*_*l*_ and takes different forms for *γ*_*l*_ = 1 and *γ*_*l*_ = 0 in BayesD*π*. This modification allows MCMC iteration of MCBayes to be performed with only Gibbs sampling.

In the simultaneous analysis of multiple traits for constructing a GBV prediction model, the computational burden greatly increases depending on the number of analyzed traits in comparison with single-trait analysis. We developed a variational approximation procedure, varBayes, for MCBayes to reduce the computational time for multi-trait analysis. In varBayes, the joint posterior distribution of parameters was approximated by a factorized function, each component of which approximated marginal posterior distribution of each parameter and was referred to as a variational posterior. Variational posteriors were shown to be well-known distribution functions such as normal or inverse Wishart that could be derived by simple non-MCMC based numerical iteration.

In genomic selection, it is important to construct a model that enables accurate prediction for GVBs. Therefore, precise point estimation of the model parameters is more relevant rather than the construction of their posterior distributions. Accordingly, the evaluation of loss of prediction accuracy with varBayes in comparison with MCBayes would be suitable for the evaluation of approximation accuracy of varBayes. Using simulation experiments, we investigated the performance of the prediction model constructed with multi-trait analysis compared with single-trait analysis as well as the model constructed using variational approximation. Moreover, the performance of multi-trait analysis in the case of missing phenotypic records commonly occurring in the treatment of the actual data of multiple traits were evaluated based on the results of simulations. These points are discussed below including the computational time and the possible extension of prediction model considering polygenic effects.

### Increase in accuracy for GBV prediction with multi-trait analysis

We evaluated the increase in prediction accuracy with multi-trait analysis in comparison with single-trait analysis using the datasets without missing phenotypes, Data I and Data II (Table 1 and Table 2). For trait A having heritability 0.8, multi-trait analysis and single-trait analysis made no difference in the prediction accuracy with MCBayes. Therefore, the advantage of multi-trait analysis over single-trait analysis in predicting GBV is negligible for high-heritability traits. However, we anticipate the increase in accuracy with multi-trait analysis for low-heritability traits utilizing correlations with high-heritability traits. Actually, for low-heritability trait B with heritability 0.1, which has highly correlated with trait A (*ρ*_GAB_ = 0.72), prediction accuracy was increased with multi-trait analysis. As trait C was not genetically correlated with trait A, the accuracy of predicting the GBV of C was not improved with multi-trait MCBayes analysis. The accuracy of predicted GBV of trait B was also increased with multi-trait varBayes analysis in Data I and Data II in comparison with single-trait MCBayes analysis while the prediction accuracy of trait C was lower with multi-trait varBayes analysis (Table 1 and Table 2). The genetic correlations between traits A and B were better estimated with predicted GBVs in multi-trait analysis than in single-trait analysis as shown in Table 5. Therefore, it can be concluded that low-heritability traits (heritability around 0.1) are better predicted for GBVs utilizing their correlations with high-heritability traits (heritability around 0.8), if any, with multi-trait analysis. However, the benefit of multi-trait analysis would be subtle for high-heritability traits. A similar finding was also reported in [13]. For uncorrelated low-heritability traits such as trait C, it is likely that the prediction using multi-trait analysis with *π* varied and inferred is less effective in comparison with single-trait analysis. Therefore, multi-trait analysis with *π* fixed at 0, which allows highly accurate prediction for correlated traits while retaining prediction accuracy comparable with single-trait analysis for uncorrelated low-heritability traits, would be a suitable method of choice for multi-trait genomic selection.

### Approximation accuracy of variational procedure for MCMC estimation

Generally, constructing a GBV prediction model with MCMC estimation based on genotypic records for tens of thousands of SNPs and phenotypes for hundreds of individuals requires considerable computational time even for single-trait cases. Much more computational burden would be imposed in constructing a model in multi-trait analysis, depending on the number of traits of interest. Therefore, we proposed a computationally cost-effective method, varBayes, approximating MCMC based method, MCBayes, using a variational approximation procedure.

Simulation experiments showed that the prediction accuracy was lower with varBayes than with MCBayes in multi-trait analysis but the rate of loss of accuracy was not remarkable and was less than 10 percent for traits A and B under the same setting of *π* while it was greater for C (Table 1 and Table 2). The prediction accuracy for trait B correlated with high-heritability trait A was still higher with multi-trait varBayes analysis than with single-trait MCBayes analysis indicating that varBayes could well utilize the information on the correlation structure in multiple traits. Actually, multi-trait varBayes analysis could better capture the genetic correlation between A and B than single-trait MCbayes analysis (Table 5).

The computational time was greatly reduced for multi-trait varBayes analysis in comparison with multi-trait MCBayes analysis. We carried out all computations using a Fortran program written to implement multiple-trait analysis on a computer having two CPUs each with a quad-core processor (Intel Xeon 2.4GHz). In Data I, where 100 replicates of datasets each including genotypes of 1010 SNPs for 1000 individuals were simulated, varBayes took only 12 minutes with *π* fixed and 22 minutes with *π* varied for 100 times of model constructions while the computational time for MCBayes was respectively 440 and 435 minutes. Therefore, the average computational time required to construct a prediction model for each dataset in Data I was less than 15 seconds for varBayes and was more than 4 minutes for MCBayes. For a larger data, Data II, that included 20 replicates of datasets each consisting of genotypes of 10100 SNPs for 1000 individuals, the computational time was 21 and 19 minutes for varBayes with *π* fixed and *π* varied, respectively, and the time for MCBayes were increased to more than 12 hours with the average computational times for each model construction being about 1 minute for varBayes and more than 30 minutes for MCBayes. In cross-validation process in which a total of one thousand repetitions of model constructions were performed in Data I, the computational time was about 3days with MCBayes while varBayes completed the analysis within only 4 hours.

Taking computational time and prediction accuracy into account, varBayes is considered a useful method for multi-trait genomic selection, which can rapidly construct a prediction model that is less accurate than that with the MCMC-based method for multi-trait analysis, but is more accurate than that with single-trait analysis for correlated traits. The usefulness of varBayes would be more remarkable for simultaneous prediction of GBVs of a large number of traits based on a huge number of SNPs where the application of an MCMC-based method might be prohibited.

### Multi-trait analysis of dataset with missing phenotypes

In Data III, we simulated the datasets under the same condition as Data I except that some phenotypes were assumed to be unobserved. In short, we assumed that phenotypes of traits A, B and C were not available for 200, 500 and 500 individuals, respectively, in a total of 1000 individuals with only 100 individuals having the phenotypes of all three traits. In multi-trait analysis, missing phenotypes of individuals can be estimated with their observed phenotypes of other traits using (18), which indicates that residual effects of missing phenotypes can be restored from those of observed phenotypes. When the model fitting is successful for observed phenotypes, the residual effects of the phenotypes are well estimated by subtracting SNP effects and other fixed effects from the phenotypic effects, and those of missing phenotypes are suitably obtained by (18) utilizing the environmental correlation (covariance) between observed and missing phenotypes. Therefore, by assuming non-zero environmental correlation between traits (*ρ*_EAB_ = 0.1, *ρ*_EAC_ = 0.2, *ρ*_EBC_ = 0.3), the loss of prediction accuracy caused by missing phenotypes was anticipated to be less with multi-trait analysis than with single-trait analysis. We expected that, in Data III, the prediction accuracy of trait C, environmentally correlated with trait A (*ρ*_EAC_ = 0.2), was maintained higher in the presence of missing records for some phenotypes using multi-trait analysis in comparison with single-trait analysis. However, the rate of loss for the prediction accuracy for trait C was similar between multi-trait and single-trait analyses as seen in the comparison of the prediction accuracies between Data I and Data III (Tables 1 and 3). The utility of implementation of (18) for imputing missing phenotypes in the process of multi-trait model construction remains unclear in the settings of simulation adopted here.

### Model extension by including polygenic effects

We can modify the Bayesian model (1) by including polygenic effects as follows;

(19) $$y_{i}=X_{i}b+\sum_{l=1}^{N}\gamma_{l}u_{\mathrm{il}}g_{l}+v_{i}+e_{i}\text{,}$$

where **v**_*i*_ is a vector of polygenic effects for multiple traits and assumed to follow a multivariate normal distribution, **v**_*i*_ ~ *N*(**0**, **Σ**_*v*_) with **Σ**_*v*_ being a variance-covariance matrix of polygenic effects. The polygenic effects for all individuals of a training population and a test population are collectively denoted by **V**, then the variance-covariance matrix of **V** can be expressed as **A** ⊗ **Σ**_*v*_, where **A** is additive genetic relationship matrix for analyzed individuals, computed from the information of pedigree or markers. When the low-density markers are used for the analysis, a considerable portion of genetic effects could not be captured by markers. Accordingly, if pedigree information is available, inclusion of polygenic effects estimated based on pedigree is beneficial in predicting breeding values for genomic selection. The revised model (19) can be similarly treated by MCBayes and varBayes as the model (1), where estimation steps for additional covariates and parameters, **V** and **Σ**_*v*_, can be easily implemented in the procedures of Bayesian multi-trait model construction using MCMC iteration and variational approximation. When no pedigree information is available, **A** is computed from marker information as a genomic relationship matrix. However, the model (19) includes all available markers as covariates, resulting in redundantly using the same marker information in the model fitting. In the availability of high-density SNP markers, which is the case for some species of animals and plants currently, the genetic relationships between individuals can be well captured by markers themselves in the multi-locus model (1), the benefit from the inclusion of the polygenic effects in the model would be subtle [31].

## Conclusion

In this study, we described a statistical model for Bayesian simultaneous prediction of GBVs in genomic selection targeting multiple traits and devised an MCMC-based method and a computationally cost-effective method utilizing the variational approximation procedure, referred to as MCBayes and varBayes, respectively, to estimate parameters included in the model. The results of simulation experiments showed that the multi-trait analysis that could utilize the correlation structure between traits allowed more accurate prediction of GBVs for correlated traits compared to single-trait analysis that treated each trait separately, where, for low-heritability traits correlated with high-heritability traits, the prediction accuracy for GBVs was remarkably improved with multi-trait analysis. Although the prediction accuracy with varBayes was lower than that with MCBayes in multi-trait analysis, the rate of loss in accuracy was moderate and the accuracy for correlated low-heritability traits was still higher with varBayes analysis compared to single-trait analysis. Considering the benefit of greatly reduced computational time, varBayes was considered to be a practical method for predicting GBVs in multi-trait genomic selection.

## Competing interests

The authors declare that they have no competing interests.

## Authors' contributions

TH devised Bayesian prediction methods for the genomic selection of multiple traits, developed a program for simulations and drafted the manuscript. HI assisted in developing a program and drafted the final manuscript. Both authors read and approved the final manuscript.

## Supplementary Material

Additional file 1 Derivation of full conditional posterior distributions of parameters in a statistical model. Click here for file

Additional file 2 Derivation of variational posteriors for parameters in a statistical model. Click here for file
