# Supplementary material for: A Bayesian method and its variational approximation for prediction of genomic breeding values in multiple traits
Source: BMC Bioinformatics. 2013 Jan 31;14:34. doi: 10.1186/1471-2105-14-34 (PMC3574034; doi:10.1186/1471-2105-14-34)
Supplement: Additional file 1 — Derivation of full conditional posterior distributions of parameters in a statistical model. [file 1471-2105-14-34-S1.pdf]

Appendix A

Derivation of full conditional posterior distributions of parameters in a statistical model

We can derive a full conditional posterior distribution for each of parameters $\boldsymbol{\theta}$=($\mathbf{b}$, $\mathbf{g}_l$, γ_l, $\boldsymbol{\Sigma}_e$, $\boldsymbol{\Sigma}_{gl}$) (l=1,2,…,N) which is required for Gibbs sampling from a joint posterior distribution of $\boldsymbol{\theta}$, g($\boldsymbol{\theta}$|ν,$\mathbf{Y}$, $\mathbf{U}$), given in (4). To determine the form of each full conditional posterior distribution we regard a joint posterior distribution as a function of a relevant parameter with the other parameters treated as constants. The full conditional posterior distribution of each parameter is denoted as p(.|else) and is described below.

Full conditional posterior distributions of b and $\boldsymbol{\Sigma}_e$

We can write (4) as,

$$g(\boldsymbol{\theta}|\nu,\mathbf{Y},\mathbf{U}) \propto \exp\{-\frac{1}{2}\sum_{i=1}^{n}(\mathbf{y}_i-\mathbf{X}_i\mathbf{b}-\sum_{l=1}^{N}\gamma_l u_{il}\mathbf{g}_l)'\boldsymbol{\Sigma}_e^{-1}(\mathbf{y}_i-\mathbf{X}_i\mathbf{b}-\sum_{l=1}^{N}\gamma_l u_{il}\mathbf{g}_l)\},$$

which is regarded as a function of parameter $\mathbf{b}$ and further expressed as

$$p(\mathbf{b}|\text{else}) \propto |\boldsymbol{\Sigma}_b|^{-n/2}\exp\{-\frac{1}{2}(\mathbf{b}-\boldsymbol{\mu}_b)'\boldsymbol{\Sigma}_b^{-1}(\mathbf{b}-\boldsymbol{\mu}_b)\},$$

where

$$\boldsymbol{\Sigma}_b=\sum_{i=1}^{n}(\mathbf{X}_i'\boldsymbol{\Sigma}_e^{-1}\mathbf{X}_i)^{-1} \text{ and } \boldsymbol{\mu}_b=\sum_{i=1}^{n}(\mathbf{X}_i'\boldsymbol{\Sigma}_e^{-1}\mathbf{X}_i)^{-1}\sum_{i=1}^{n}\mathbf{X}_i'\boldsymbol{\Sigma}_e^{-1}(\mathbf{y}_i-\sum_{l=1}^{N}\gamma_l u_{il}\mathbf{g}_l).$$

Therefore, the full conditional posterior distribution of $\mathbf{b}$ is a T-variate normal distribution with a mean vector $\boldsymbol{\mu}_b$ and variance covariance matrix $\boldsymbol{\Sigma}_b$.

On the other hand, we modified (4), by regarding it as a function of $\boldsymbol{\Sigma}_e$, as

g($\boldsymbol{\theta}$|ν,**Y**,**U**)

$$\propto |\boldsymbol{\Sigma}_e|^{-n/2} \exp[-\frac{1}{2}\mathrm{tr}\{\sum_{i=1}^{n}(\mathbf{y}_i - \mathbf{X}_i\mathbf{b} - \sum_{l=1}^{N}\gamma_l u_{il}\mathbf{g}_l)(\mathbf{y}_i - \mathbf{X}_i\mathbf{b} - \sum_{l=1}^{N}\gamma_l u_{il}\mathbf{g}_l)'\boldsymbol{\Sigma}_e^{-1}\}] .$$

Accordingly, the full conditional posterior distribution of $\boldsymbol{\Sigma}_e$, $p(\boldsymbol{\Sigma}_e$ |else), was an inverse Wishart distribution, $\mathrm{IW}_T(\nu_e,\mathbf{S}_e)$, with degree of freedom $\nu_e=n-T-1$ and scale parameter

$$\mathbf{S}_e = \sum_{i=1}^{n}(\mathbf{y}_i - \mathbf{X}_i\mathbf{b} - \sum_{l=1}^{N}\gamma_l u_{il}\mathbf{g}_l)(\mathbf{y}_i - \mathbf{X}_i\mathbf{b} - \sum_{l=1}^{N}\gamma_l u_{il}\mathbf{g}_l)'.$$

Full conditional posterior distributions of $\mathbf{g}_l$ and γ_l

We obtain a joint full conditional posterior distribution function of $\mathbf{g}_l$ and γ_l , $p(\mathbf{g}_l,\gamma_l|\text{else})$, from (4) as follows;

$$p(\mathbf{g}_l, \gamma_l\,|\text{else}) \propto \exp\{-\frac{1}{2}\sum_{i=1}^{n}(\mathbf{y}_i^{\#} - \gamma_l u_{il}\mathbf{g}_l)\boldsymbol{\Sigma}_e^{-1}(\mathbf{y}_i^{\#} - \gamma_l u_{il}\mathbf{g}_l)\}$$

$$\times\{(1-\pi)\,|\boldsymbol{\Sigma}_{gl}|^{-1/2}\exp(-\frac{1}{2}\mathbf{g}_l{}'\boldsymbol{\Sigma}_{gl}^{-1}\mathbf{g}_l)\}^{\gamma_l}\{\pi\delta(\mathbf{0})\}^{1-\gamma_l},$$

where $\mathbf{y}_i^{\#} = \mathbf{y}_i - \mathbf{X}_i\mathbf{b} - \sum_{m\neq l}^{N}\gamma_m u_{im}\mathbf{g}_m$ means the residual of $\mathbf{y}_i$ subtracted by all effects except of $\mathbf{g}_l$. We can further express $p(\mathbf{g}_l, \gamma_l$ |else) as

$p(\mathbf{g}_l, \gamma_l$ |else)

$$\propto [(1-\pi)\,|\mathbf{R}_{gl}|^{1/2}|\boldsymbol{\Sigma}_{gl}^{-1}|^{1/2}\exp(\frac{1}{2}\boldsymbol{\mu}_{gl}{}'\mathbf{R}_{gl}^{-1}\boldsymbol{\mu}_{gl})\phi(\mathbf{g}_l\,|\,\boldsymbol{\mu}_{gl},\mathbf{R}_{gl})]^{\gamma_l}\{\pi\delta(\mathbf{0})\}^{1-\gamma_l}, \quad \text{(A1)}$$

where $\boldsymbol{\mu}_{gl} = (\boldsymbol{\Sigma}_{gl}^{-1} + \sum_{i=1}^{n}u_{il}^{2}\boldsymbol{\Sigma}_e^{-1})^{-1}\boldsymbol{\Sigma}_e^{-1}\sum_{i=1}^{n}u_{il}\mathbf{y}_i^{\#}$, $\mathbf{R}_{gl} = (\boldsymbol{\Sigma}_{gl}^{-1} + \sum_{i=1}^{n}u_{il}^{2}\boldsymbol{\Sigma}_e^{-1})^{-1}$ and

$\phi(\mathbf{g}_l|\boldsymbol{\mu}_{gl},\mathbf{R}_{gl})$ is a density function of T-variate normal with mean vector $\boldsymbol{\mu}_{gl}$ and variance covariance matrix $\mathbf{R}_{gl}$. From (A1), full conditional posterior distribution of γ_l is obtained by integrating out $\mathbf{g}_l$;

$p(\gamma_l=1|\text{else})=\int p(\mathbf{g}_l,\gamma_l=1|\text{else})d\mathbf{g}_l$ and $p(\gamma_l=0|\text{else})=\int p(\mathbf{g}_l,\gamma_l=0|\text{else})d\mathbf{g}_l$.

Accordingly, we can show that

$$p(\gamma_l=1|\text{else})=\frac{\int p(\mathbf{g}_l,\gamma_l=1|\text{else})d\mathbf{g}_l}{\int p(\mathbf{g}_l,\gamma_l=1|\text{else})d\mathbf{g}_l+\int p(\mathbf{g}_l,\gamma_l=0|\text{else})d\mathbf{g}_l}$$

$$=\frac{(1-\pi)|\mathbf{R}_{gl}|^{1/2}|\boldsymbol{\Sigma}_{gl}|^{-1/2}\exp(\boldsymbol{\mu}_{gl}'\mathbf{R}_{gl}^{-1}\boldsymbol{\mu}_{gl}/2)}{(1-\pi)|\mathbf{R}_{gl}|^{1/2}|\boldsymbol{\Sigma}_{gl}|^{-1/2}\exp(\boldsymbol{\mu}_{gl}'\mathbf{R}_{gl}^{-1}\boldsymbol{\mu}_{gl}/2)+\pi}$$

and

$$p(\gamma_l=0|\text{else})=\frac{\int p(\mathbf{g}_l,\gamma_l=0|\text{else})d\mathbf{g}_l}{\int p(\mathbf{g}_l,\gamma_l=1|\text{else})d\mathbf{g}_l+\int p(\mathbf{g}_l,\gamma_l=0|\text{else})d\mathbf{g}_l}$$

$$=\frac{\pi}{(1-\pi)|\mathbf{R}_{gl}|^{1/2}|\boldsymbol{\Sigma}_{gl}|^{-1/2}\exp(\boldsymbol{\mu}_{gl}'\mathbf{R}_{gl}^{-1}\boldsymbol{\mu}_{gl}/2)+\pi}.$$

The value of γ_l is sampled from a binomial probability with an occurrence probability $p(\gamma_l=1|\text{else})$. Given γ_l, it can be shown from (A1) that the full conditional posterior distribution of $\mathbf{g}_l$, $p(\mathbf{g}_l\,|\text{else})$, is a mixture distribution of a normal distribution $N(\boldsymbol{\mu}_{gl},\mathbf{R}_{gl})$ and a probability distribution concentrating a total mass at 0 $\delta(\mathbf{0})$ with a mixture probability of $p(\gamma_l=1|\text{else})$. Accordingly, the value of $\mathbf{g}_l$ is drawn from the following distribution;

$$\mathbf{g}_l\sim\begin{cases}N(\boldsymbol{\mu}_{gl},\mathbf{R}_{gl}) & (\gamma_l=1)\\ 0 & (\gamma_l=0)\end{cases}.$$

Full conditional posterior distribution of $\boldsymbol{\Sigma}_{gl}$

We modify (4) as a function of $\boldsymbol{\Sigma}_{gl}$ (l=1,2,…,N);

$$g(\boldsymbol{\theta}|\nu,\mathbf{Y},\mathbf{U}) \propto |\boldsymbol{\Sigma}_{gl}|^{-n\gamma_l/2}\exp\{-\frac{1}{2}\mathrm{tr}(\gamma_l\mathbf{g}_l\mathbf{g}_l'\boldsymbol{\Sigma}_{gl}^{-1})\}|\boldsymbol{\Sigma}_{gl}|^{-(\nu+T+1)/2}\exp(-\frac{1}{2}\mathrm{tr}\mathbf{S}\boldsymbol{\Sigma}_{gl}^{-1}) ,$$

from which a full conditional posterior distribution of $\boldsymbol{\Sigma}_{gl}$, $p(\boldsymbol{\Sigma}_{gl}\,|\text{else})$, is shown to be an inverse Wishart distribution, $\mathrm{IW}_T(\nu+\gamma_l,\ \gamma_l\mathbf{g}_l\mathbf{g}_l'+\mathbf{S})$, and is presented as

$$p(\boldsymbol{\Sigma}_{gl}\,|\text{else}) \propto |\boldsymbol{\Sigma}_{gl}|^{-(\nu+\gamma_l+T+1)/2}\exp[-\frac{1}{2}\mathrm{tr}\{(\gamma_l\mathbf{g}_l\mathbf{g}_l'+\mathbf{S})\boldsymbol{\Sigma}_{gl}^{-1}\}].$$

Full conditional posterior distribution of S

By arranging the terns including **S** in (4) we can obtain a full conditional posterior distribution of **S** as

$$p(\mathbf{S}\,|\text{else}) \propto |\mathbf{S}|^{N\nu/2}\exp\{-\frac{1}{2}\mathrm{tr}(\sum_{l=1}^{N}\boldsymbol{\Sigma}_{gl}^{-1}\mathbf{S})\},$$

which is a Wishart distribution with degree of freedom $N\nu+T+1$ and scale parameter

$$\{\textstyle\sum_{l=1}^{N}\boldsymbol{\Sigma}_{gl}^{-1}\}^{-1} .$$

Full conditional posterior distribution of π

When a prior probability that a SNP has zero effect, π, is inferred, π can be sampled from a beta distribution given as

$$p(\pi\,|\text{else}) \propto (1-\pi)^{\sum_{l=1}^{N}\gamma_l}\pi^{N-\sum_{l=1}^{N}\gamma_l},$$

which is derived from (4).
