# Supplementary material for: A Bayesian method and its variational approximation for prediction of genomic breeding values in multiple traits
Source: BMC Bioinformatics. 2013 Jan 31;14:34. doi: 10.1186/1471-2105-14-34 (PMC3574034; doi:10.1186/1471-2105-14-34)
Supplement: Additional file 2 — Derivation of variational posteriors for parameters in a statistical model. [file 1471-2105-14-34-S2.pdf]

Appendix B

Derivation of variational posteriors for parameters in a statistical model

We can derive a variational posterior for each of parameters $\boldsymbol{\theta}$=($\mathbf{b}$, $\mathbf{g}_l$, γ_l, $\boldsymbol{\Sigma}_e$, $\boldsymbol{\Sigma}_{gl}$, $\mathbf{S}$) (l=1,2,…,N) from (4) and (5) following a similar manner as used for the derivation of full conditional posteriors in Gibbs sampling (Additional file 1). It can be shown from (4) that log{g($\boldsymbol{\theta}$| ν,$\mathbf{Y}$, $\mathbf{U}$)}is presented as

log{g($\boldsymbol{\theta}$| ν,$\mathbf{Y}$, $\mathbf{U}$)}

$$=C-\frac{n}{2}\log|\boldsymbol{\Sigma}_e|-\frac{1}{2}\sum_{i=1}^{n}\mathbf{y}_i{}^*\boldsymbol{\Sigma}_e{}^{-1}\mathbf{y}_i{}^*+\sum_{l=1}^{N}\gamma_l\{\log(1-\pi)-\frac{1}{2}\log|\boldsymbol{\Sigma}_{gl}|-\frac{1}{2}\mathbf{g}_l{}'\boldsymbol{\Sigma}_{gl}{}^{-1}\mathbf{g}_l\}$$

$$+\sum_{l=1}^{N}(1-\gamma_l)\{\log\pi+\log\delta(\mathbf{0})\}+\frac{N\nu}{2}\log|\mathbf{S}|-\frac{\nu+T+1}{2}\sum_{l=1}^{N}\log|\boldsymbol{\Sigma}_{gl}|-\frac{1}{2}\mathrm{tr}(\mathbf{S}\sum_{l=1}^{N}\boldsymbol{\Sigma}_{gl}{}^{-1}),$$

(B1)

where C means a constant and $\mathbf{y}_i$*= $\mathbf{y}_i$ -$\mathbf{X}_i\mathbf{b}$ - $\sum_{l=1}^{N}\gamma_l u_{il}\mathbf{g}_l$.

Hereafter, we denote the variational posterior with q(.) and the expectation taken with respect to the variational posterior with E(.) for each parameter. Based on (5) and (B1), the derivation of variational posterior is described for each parameter in the following.

Variational posteriors of b and $\boldsymbol{\Sigma}_e$

We can write (B1) as a function of parameter $\mathbf{b}$,

$$\log\{g(\boldsymbol{\theta}|\nu,\mathbf{Y},\mathbf{U})\}=C-\frac{1}{2}\sum_{i=1}^{n}(\mathbf{y}_i-\mathbf{X}_i\mathbf{b}-\sum_{l=1}^{N}\gamma_l u_{il}\mathbf{g}_l)'\boldsymbol{\Sigma}_e{}^{-1}(\mathbf{y}_i-\mathbf{X}_i\mathbf{b}-\sum_{l=1}^{N}\gamma_l u_{il}\mathbf{g}_l).$$

This is a quadratic function of $\mathbf{b}$ and can be arranged as

$$\log\{g(\boldsymbol{\theta}|\nu,\mathbf{Y},\mathbf{U})\}=C-\frac{1}{2}(\mathbf{b}-\boldsymbol{\mu}_b)'\boldsymbol{\Sigma}_b{}^{-1}(\mathbf{b}-\boldsymbol{\mu}_b),$$

where

$$\boldsymbol{\Sigma}_b = \sum_{i=1}^{n}(\mathbf{X}_i{}'\boldsymbol{\Sigma}_e{}^{-1}\mathbf{X}_i)^{-1} \quad \text{and} \quad \boldsymbol{\mu}_b = \sum_{i=1}^{n}(\mathbf{X}_i{}'\boldsymbol{\Sigma}_e{}^{-1}\mathbf{X}_i)^{-1}\sum_{i=1}^{n}\mathbf{X}_i{}'\boldsymbol{\Sigma}_e{}^{-1}(\mathbf{y}_i - \sum_{l=1}^{N}\gamma_l u_{il}\mathbf{g}_l)$$

as already described in additional file 1. From (5), we obtain a variational posterior of **b** as

$$q(\mathbf{b}) \propto |\hat{\boldsymbol{\Sigma}}_b|^{-1/2}\exp\{-\frac{1}{2}(\mathbf{b}-\hat{\boldsymbol{\mu}}_b)'\hat{\boldsymbol{\Sigma}}_b{}^{-1}(\mathbf{b}-\hat{\boldsymbol{\mu}}_b)\}$$

which is a T-variate normal distribution having a mean vector $\hat{\boldsymbol{\mu}}_b$ and a variance covariance matrix $\hat{\boldsymbol{\Sigma}}_b$ with $\hat{\boldsymbol{\mu}}_b$ and $\hat{\boldsymbol{\Sigma}}_b$ being $\boldsymbol{\mu}_b$ and Σ_b substituted for Σ_e^{-1} and $\gamma_l\mathbf{g}_l$ by their expectations taken with respect to their variational posteriors as shown in (6).

Similarly, (B1) is regarded as a function of Σ_e and can be expressed as

$$\log\{g(\theta|\, \nu, \mathbf{Y}, \mathbf{U})\} = C - \frac{n}{2}\log|\boldsymbol{\Sigma}_e| - \frac{1}{2}\sum_{i=1}^{n}\mathbf{y}_i{}^{*}\boldsymbol{\Sigma}_e{}^{-1}\mathbf{y}_i{}^{*},$$

from which, using (5), it is shown that $q(\Sigma_e)$ is an inverse Wishart distribution of a form

$$q(\Sigma_e) = \mathrm{IW}_T(n-T-1, \sum_{i=1}^{n}\hat{\mathbf{y}}_i{}^{*}\hat{\mathbf{y}}_i{}^{*\prime}) \propto |\boldsymbol{\Sigma}_e|^{-n/2}\exp\{-\frac{1}{2}\mathrm{tr}(\sum_{i=1}^{n}\hat{\mathbf{y}}_i{}^{*}\hat{\mathbf{y}}_i{}^{*}\boldsymbol{\Sigma}_e{}^{-1})\},$$

where

$$\hat{\mathbf{y}}_i{}^{*} = \mathbf{y}_i - \mathbf{X}_i\mathrm{E}(\mathbf{b}) - \sum_{l=1}^{N}u_{il}\mathrm{E}(\gamma_l\mathbf{g}_l)$$

as seen in (7).

Variational posteriors of $\mathbf{g}_l$ and γ_l

For $\mathbf{g}_l$ and γ_l (l=1,2,…,N), we firstly consider a joint variational posterior. From (B1), we arrange the terms including $\mathbf{g}_l$ and γ_l and use (5) to obtain $q(\mathbf{g}_l, \gamma_l)$ in a similar way as used in the derivation of (A1);

$q(\mathbf{g}_l, \gamma_l)$

$$\propto [(1-\pi)\,|\,\mathbf{V}_{gl}\,|^{1/2}|\,\mathrm{E}(\boldsymbol{\Sigma}_{gl}{}^{-1})\,|^{1/2}\,\exp(\frac{1}{2}\hat{\mathbf{g}}_l{}'\mathbf{V}_{gl}{}^{-1}\hat{\mathbf{g}}_l)\phi(\mathbf{g}_l\,|\,\hat{\mathbf{g}}_l,\mathbf{V}_{gl})]^{\gamma_l}\{\pi\delta(\mathbf{0})\}^{1-\gamma_l}\text{ , (B2)}$$

where

$$\hat{\mathbf{g}}_l = \{\mathrm{E}(\boldsymbol{\Sigma}_{gl}{}^{-1}) + \sum_{i=1}^{n} u_{il}{}^{2}\mathrm{E}(\boldsymbol{\Sigma}_e{}^{-1})\}^{-1}\mathrm{E}(\boldsymbol{\Sigma}_e{}^{-1})\sum_{i=1}^{n} u_{il}\{\mathbf{y}_i - \mathbf{X}_i\mathrm{E}(\mathbf{b}) - \sum_{m\neq l}^{N} u_{im}\mathrm{E}(\gamma_m\mathbf{g}_m)\}$$

and

$$\mathbf{V}_{gl} = \{\mathrm{E}(\boldsymbol{\Sigma}_{gl}{}^{-1}) + \sum_{i=1}^{n} u_{il}{}^{2}\mathrm{E}(\boldsymbol{\Sigma}_e{}^{-1})\}^{-1}.$$

Subsequently, the variational posterior of γ_l is obtained by integrating out $\mathbf{g}_l$ from $q(\mathbf{g}_l,\gamma_l)$;

$q(\gamma_l=1)=\int q(\mathbf{g}_l,\gamma_l=1)d\mathbf{g}_l$ and $q(\gamma_l=0)=\int q(\mathbf{g}_l,\gamma_l=0)d\mathbf{g}_l$,

which can be represented as

$$q(\gamma_l=1)=\frac{\int q(\mathbf{g}_l,\gamma_l=1)d\mathbf{g}_l}{\int q(\mathbf{g}_l,\gamma_l=1)d\mathbf{g}_l+\int q(\mathbf{g}_l,\gamma_l=0)d\mathbf{g}_l}$$

$$=\frac{(1-\pi)\,|\,\mathbf{V}_{gl}\,|^{1/2}|\,\mathrm{E}(\boldsymbol{\Sigma}_{gl})\,|^{-1/2}\,\exp(\hat{\mathbf{g}}_l{}'\mathbf{V}_{gl}{}^{-1}\hat{\mathbf{g}}_l/2)}{(1-\pi)\,|\,\mathbf{V}_{gl}\,|^{1/2}|\,\mathrm{E}(\boldsymbol{\Sigma}_{gl})\,|^{-1/2}\,\exp(\hat{\mathbf{g}}_l{}'\mathbf{V}_{gl}{}^{-1}\hat{\mathbf{g}}_l/2)+\pi}$$

and

$$q(\gamma_l=0)=\frac{\int q(\mathbf{g}_l,\gamma_l=0)d\mathbf{g}_l}{\int q(\mathbf{g}_l,\gamma_l=1)d\mathbf{g}_l+\int q(\mathbf{g}_l,\gamma_l=0)d\mathbf{g}_l}$$

$$=\frac{\pi}{(1-\pi)\,|\,\mathbf{V}_{gl}\,|^{1/2}|\,\mathrm{E}(\boldsymbol{\Sigma}_{gl})\,|^{-1/2}\,\exp(\hat{\mathbf{g}}_l{}'\mathbf{V}_{gl}{}^{-1}\hat{\mathbf{g}}_l/2)+\pi}.$$

Using $q(\gamma_l=1)$ and $q(\gamma_l=0)$, from (B2), the variational posterior of $\mathbf{g}_l$ is given as

$q(\mathbf{g}_l)=q(\mathbf{g}_l, \gamma_l=1)+ q(\mathbf{g}_l, \gamma_l=0)$

$= q(\gamma_l\!=\!1)\ \phi(\mathbf{g}_l \mid \hat{\mathbf{g}}_l, \mathbf{V}_{gl}) + q(\gamma_l\!=\!0)\delta(\mathbf{0})$,

which is a mixture distribution of a normal distribution $N(\hat{\mathbf{g}}_l, \mathbf{V}_{gl})$ and a probability distribution concentrating a total mass on zero $\delta(\mathbf{0})$ with a mixture probability of $q(\gamma_l\!=\!1)$.

Variational posterior of Σ_{gl}

We modify (B1) by arranging the terms including Σ_{gl} (l=1,2,…,N) as

$$\log\{g(\theta|\nu,\mathbf{Y},\mathbf{U})\}=C-\frac{\nu+\gamma_l+T+1}{2}\log|\mathbf{\Sigma}_{gl}|-\frac{1}{2}\mathrm{tr}[\{\mathrm{E}(\gamma_l\mathbf{g}_l\mathbf{g}_l')+\mathrm{E}(\mathbf{S})\}\mathbf{\Sigma}_{gl}^{-1}].$$

From (5), the variational posterior of Σ_{gl} is expressed as

$$q(\Sigma_{gl}) = \mathrm{IW}_T(\nu+\gamma_l,\ \mathrm{E}(\gamma_l\mathbf{g}_l\mathbf{g}_l')+\mathrm{E}(\mathbf{S}))$$

$$\propto|\mathbf{\Sigma}_{gl}|^{-(\nu+\gamma_l+T+1)/2}\ \exp(-\frac{1}{2}\mathrm{tr}[\{\mathrm{E}(\gamma_l\mathbf{g}_l\mathbf{g}_l')+\mathrm{E}(\mathbf{S})\}\mathbf{\Sigma}_{gl}^{-1}])$$

Variational posterior of S

By arranging the terns including **S** in (B1) we can obtain

$$\log\{g(\theta|\nu,\mathbf{Y},\mathbf{U})\}=C+\frac{N\nu}{2}\log|\mathbf{S}|-\frac{1}{2}\mathrm{tr}(\sum_{l=1}^{N}\mathbf{\Sigma}_{gl}^{-1}\mathbf{S}),$$

from which the variational posterior of **S** can be expressed, using (5), as,

$$q(\mathbf{S})\propto|\mathbf{S}|^{N\nu/2}\ \exp[-\frac{1}{2}\mathrm{tr}\{\sum_{l=1}^{N}\mathrm{E}(\mathbf{\Sigma}_{gl}^{-1})\mathbf{S}\}].$$

This distribution is a Wishart distribution with degree of freedom $N\nu+T+1$ and scale parameter $\{\sum_{l=1}^{N}\mathrm{E}(\mathbf{\Sigma}_{gl}^{-1})\}^{-1}$.

Variational posterior of π

When a prior probability that a SNP has zero effect, π, is inferred, the variational posterior of π can be expressed as the following beta distribution from (B1) and (5);

$$q(\pi) \propto (1-\pi)^{\sum_{l=1}^{N} \mathrm{E}(\gamma_l)} \pi^{N-\sum_{l=1}^{N} \mathrm{E}(\gamma_l)},$$

where it should be noted that $\mathrm{E}(\gamma_l) = q(\gamma_l = 1)$.
